# Sleep and perivascular spaces in the middle‐aged and elderly population

**DOI:** 10.1111/jsr.13485

**Published:** 2021-09-22

**Authors:** Thom S. Lysen, Pinar Yilmaz, Florian Dubost, M. Arfan Ikram, Marleen de Bruijne, Meike W. Vernooij, Annemarie I. Luik

**Affiliations:** ^1^ Department of Epidemiology Erasmus MC University Medical Centre Rotterdam the Netherlands; ^2^ Department of Radiology and Nuclear Medicine Erasmus MC University Medical Centre Rotterdam the Netherlands; ^3^ Biomedical Imaging Group Rotterdam Department of Radiology and Nuclear Medicine Erasmus MC University Medical Centre Rotterdam the Netherlands; ^4^ Department of Neurology Erasmus MC University Medical Centre Rotterdam the Netherlands; ^5^ Department of Computer Science University of Copenhagen Copenhagen Denmark

**Keywords:** community‐dwelling, epidemiology, glymphatic, paravascular, Virchow‐Robin, VRS

## Abstract

Sleep has been hypothesised to facilitate waste clearance from the brain. We aimed to determine whether sleep is associated with perivascular spaces on brain magnetic resonance imaging (MRI), a potential marker of impaired brain waste clearance, in a population‐based cohort of middle‐aged and elderly people. In 559 participants (mean [*SD*] age 62 [6] years, 52% women) from the population‐based Rotterdam Study, we measured total sleep time, sleep onset latency, wake after sleep onset and sleep efficiency with actigraphy and polysomnography. Perivascular space load was determined with brain MRI in four regions (centrum semiovale, basal ganglia, hippocampus, and midbrain) via a validated machine learning algorithm using T2‐weighted MR images. Associations between sleep characteristics and perivascular space load were analysed with zero‐inflated negative binomial regression models adjusted for various confounders. We found that higher actigraphy‐estimated sleep efficiency was associated with a higher perivascular space load in the centrum semiovale (odds ratio 1.10, 95% confidence interval 1.04–1.16, *p* = 0.0008). No other actigraphic or polysomnographic sleep characteristics were associated with perivascular space load in other brain regions. We conclude that, contrary to our hypothesis, associations of sleep with perivascular space load in this middle‐aged and elderly population remained limited to an association of a high actigraphy‐estimated sleep efficiency with a higher perivascular space load in the centrum semiovale.

## 1 INTRODUCTION

Sleep has been hypothesised to be a key driver of waste clearance from the brain (Jessen, Munk, Lundgaard, & Nedergaard, 2015; Xie et al., 2013). Brain waste clearance involves the glia‐dependent “glymphatic” system (Iliff et al., 2012). Glymphatic clearance involves fluid exchange across the perivascular spaces (PVS) that surround small blood vessels throughout the brain (Iliff et al., 2012). Sleep has been suggested to enhance such clearance (Xie et al., 2013) in animals (Brown et al., 2018; Jessen et al., 2015) and potentially also in humans (Eide, Vinje, Pripp, Mardal, & Ringstad, 2021).

Glymphatic clearance in humans may be studied through visualising PVS non‐invasively using brain magnetic resonance imaging (MRI) (Adams et al., 2015). The PVS can become enlarged, which may increase their visibility on brain MRI (Wardlaw et al., 2020). This visibility, although still a poorly understood phenomenon (Adams et al., 2015; Wardlaw et al., 2020), is increasingly thought to reflect an underlying pathological state (Brown et al., 2018), as it is associated with vascular and neurodegenerative disease (Adams et al., 2015; Wardlaw et al., 2020).

Several, mostly clinical, studies have determined whether poor sleep determines higher PVS load on MRI and found mixed results. Some sleep characteristics, such as a lower sleep efficiency (Berezuk et al., 2015; Del Brutto, Mera, Del Brutto, & Castillo, 2019) have been associated with higher PVS load, but other studies have reported no associations for those same, and other sleep characteristics (Aribisala et al., 2019; Berezuk et al., 2015; Burnett, Witte, & Ahlskog, 2014; Del Brutto, Mera, Del Brutto, & Castillo, 2020; Opel et al., 2019; Ramirez et al., 2021; Si et al., 2020; Song et al., 2017; Wang et al., 2020; Zhang et al., 2021). Studies using population‐based samples, to see if findings generalise outside of a clinical context, are scarce (Aribisala et al., 2019; Del Brutto et al., 2019, 2020). Also, associations may differ due to the use of subjective versus objective measurements (Del Brutto et al., 2019), as subjective measurements also reflect the experience of sleep and are therefore more influenced by other factors than physiological sleep alone.

Determining whether sleep is related to PVS load can help us understand a possible aetiological role of poor sleep in neurological disease, potentially providing new treatment options. We assessed the relation of objectively measured sleep through both actigraphy and polysomnography (PSG) with MRI‐assessed PVS load in four brain regions in a population‐based cohort. We hypothesised that indicators of poor sleep would be related to higher PVS load (Aribisala et al., 2019; Berezuk et al., 2015; Burnett et al., 2014; Del Brutto et al., 2019, 2020; Opel et al., 2019; Si et al., 2020; Song et al., 2017; Zhang et al., 2021).

## 2 SUBJECTS AND METHODS

### 2.1 Study setting and population

The Rotterdam Study cohort started in 1990 and aims to investigate common chronic diseases in the elderly (Ikram et al., 2020). The cohort has since been expanded twice and currently includes 14,926 participants aged ≥45 years. Examination rounds include a home interview and subsequent visits to the dedicated research centre and are repeated every 4–5 years. Since 2005, brain MRI is part of the core protocol of the Rotterdam Study.

The Rotterdam Study has been approved by the Medical Ethics Committee of the Erasmus MC (registration number MEC 02.1015) and by the Dutch Ministry of Health, Welfare and Sport (Population Screening Act WBO, license number 1071272–159521‐PG). The study was performed in accordance with the Declaration of Helsinki. All participants provided written informed consent to participate in the study and to have their information obtained from treating physicians.

Between 2011 and 2014, 2,135 participants were invited to take part in an actigraphy sub‐study (1,773 agreed, 83%), and 1,750 participants were invited to take part in a 1‐night PSG sub‐study (929 agreed, 53%). A total of 1,038 were invited to undergo both (772 agreed, 74%). No exclusion criteria were set except for being deemed able to understand instructions. In our main analysis, we included 559 individuals who had valid data on (a) actigraphy for ≥4 consecutive days, (b) PSG, and (c) a brain MRI‐scan performed within a 2‐year timeframe. Sensitivity analyses were performed in the full samples of 1,228 participants with valid actigraphy and MRI and 769 participants with valid PSG and MRI. Both of these samples include the 559 participants in the main analyses.

### 2.2 Assessment of sleep

Participants wore an actigraph (Actiwatch, model AW4, Cambridge Technology, or Geneactiv, Activinsights Ltd), measuring acceleration aggregated into activity counts per 30‐s epochs (Van Den Berg et al., 2008). We used a previously described method to ensure comparability of estimates obtained from these devices (te Lindert & Van Someren, 2013). We instructed participants to wear the actigraph for 7 days and nights around the non‐dominant wrist, to remove it only while bathing (only for Actiwatch), and to keep a daily sleep diary (Van Den Berg et al., 2008).

Sleep data per night were considered invalid if no data were recorded, if the participant had discontinued wearing the actigraph, if it coincided with daylight savings time, or if sleep diary information on bedtime and get‐up time from which time in bed was derived was invalid or missing. Within the time in bed, the algorithm estimated the assumed sleep period based on sleep onset and sleep offset, as described previously (Van Den Berg et al., 2008). It also estimated sleep versus wakefulness per 30‐s epoch using a validated algorithm with a threshold of 20 activity counts (Van Den Berg et al., 2008). The algorithm calculated total sleep time (TST; the sum of all sleep epochs within the assumed sleep period), sleep onset latency (SOL; difference between diary‐derived bedtime and estimated sleep onset), wake after sleep onset (WASO; sum of all wake epochs within the assumed sleep period) and sleep efficiency (TST/time in bed × 100%). Sleep characteristics were averaged over all available nights. Actigraphy recording duration was a median (interquartile range [IQR]) of 144 (144–168) hr.

For the at‐home 1‐night PSG, sensors were applied according to the American Association of Sleep Medicine (AASM) criteria by trained research assistants (Iber, 2007). Sensors included six electroencephalography channels (F3/A2, F4/A1, C3/A2, C4/A1, O1/A2, O2/A1), bilateral electro‐oculography, chin electromyography, electrocardiography, respiratory belts on the chest and abdomen, oximetry, and a nasal pressure transducer and oronasal thermocouple. We instructed individuals to spend the night as normally as possible, without restrictions on bedtimes, activities, or diet.

Participants signalled the times of “lights out” and getting up using the PSG equipment, from which time in bed was calculated. Missing times were extracted from the sleep diary. All recordings were manually scored by a registered PSG technologist to determine wake, non‐rapid eye movement (NREM) sleep stage 1 (N1), stage N2, stage N3, and REM sleep (Iber, 2007). We calculated TST (sum of all sleep epochs irrespective of stage), SOL (time from “lights out” to the first epoch of sleep), WASO (sum of all wake epochs between sleep onset and getting up), sleep efficiency (TST/time in bed × 100%), and sleep stage durations (sum of all epochs per stage).

### 2.3 Assessment of PVS

Brain imaging was performed on a 1.5‐Tesla (T) MRI scanner (Signa Excite II, GE Healthcare) providing T1‐contrast (T1), T2, T2‐weighted Fluid‐Attenuated Inversion Recovery (T2‐FLAIR), and T2*‐weighted gradient‐recalled‐echo (T2*) sequences.

The PVS load was automatically assessed in four pre‐defined locations: the centrum semiovale, basal ganglia, hippocampi, and midbrain. Pre‐processing, and development of the automated assessment were described in detail previously (Dubost et al., 2019). In short, pre‐processing included extracting target brain regions with “Freesurfer” on registered T1 scans and masking surroundings. The PVS were assessed using a previously developed and validated machine learning algorithm on T2 scans (Dubost et al., 2019). The algorithm was developed on visual ratings by trained raters, performed with a standardised protocol (Adams et al., 2015), defining PVS as linear, ovoid, or round‐shaped hyperintensities on T2 scans with a diameter of ≥1 mm, and <3 mm. In the visual rating, the PVS were counted in a single pre‐defined slice in the centrum semiovale (1 cm above the uppermost part of the lateral ventricles) and the basal ganglia (slice involving the anterior commissure), and in the entire structure for the hippocampus and midbrain. The machine learning algorithm was trained per region, considering that shapes and mimics of PVS may be region specific. The algorithm estimated PVS counts for each of these four regions to simulate a visual rating, providing a non‐integer value to operationalise PVS load. The algorithm showed good agreement with the visual scores, similar to inter‐observer agreement, on an independent set of scans acquired with the same scan protocol (Dubost et al., 2019).

### 2.4 Covariates

We considered the following potential confounders: age at sleep measurement, sex, education (categorised as primary, secondary/lower vocational, intermediate vocational, and higher vocational/university), the time interval between measuring sleep and MRI scanning, smoking status (never, former, current), habitual alcohol consumption (g/day), body mass index (kg/m^2^), presence of hypertension (resting blood pressure >140/90 mmHg, or use of blood pressure‐lowering medication), presence of diabetes mellitus (serum glucose level ≥7.0 mmol/L [fasting] or ≥11.1 mmol/L [non‐fasting], or use of glucose‐lowering medication), a positive history of heart disease (myocardial infarction, heart failure, or coronary re‐vascularisation procedure), the systemic immune‐inflammation index (blood‐based biomarker calculated by multiplying counts, in 10^9^/L, of platelets with granulocytes, divided by lymphocytes; van der Willik et al., 2019), and napping (categorised as yes/no per afternoon and evening using the sleep diary, and summed over the actigraphy recording [range 0–14]). Measurements were performed during the home interview or centre visits, unless stated otherwise (Licher et al., 2019).

Additionally, we determined supratentorial intracranial volume, white matter hyperintensity volume, lacunes and cortical brain infarcts, and lobar cerebral microbleeds. Volumes were segmented automatically on T1 or T2‐FLAIR scans and confirmed or corrected by trained raters (de Boer et al., 2010). Trained raters also rated cortical infarcts (lesions involving cortical grey matter with tissue loss), lacunes (subcortical lesions ≥3 mm and <15 mm), and strictly lobar microbleeds as focal parenchymal areas of low signal on T2* images not involving deep grey and white matter structures (de Boer et al., 2010).

With regard to sleep we further determined the PSG‐derived apnea–hypopnea index (AHI) and desaturation rate (PRANA, PhiTools). The AHI was automatically calculated as the number of apneas and hypopneas per hour of sleep according to AASM criteria (Iber, 2007). Similarly, desaturation rate was calculated as the number of desaturations of ≥3% from baseline per hour of sleep.

### 2.5 Statistical analysis

We associated TST, SOL, WASO, and sleep efficiency, assessed with both actigraphy and PSG, with the PVS load in four regions. All sleep characteristics were winsorised to 3 standard deviations (*SD*) from the mean, and subsequently standardised to facilitate comparison across characteristics; this was done separately for the main analysis sample, and the full actigraphy and PSG samples. We used zero‐inflated negative binomial regression models to account for excess zeros in the PVS count data (Dubost et al., 2019). Analyses were adjusted for age, sex, education, and the time interval between measuring sleep and MRI (model 1), and additionally for smoking status, habitual alcohol consumption, body mass index, hypertension, diabetes mellitus, a history of heart disease, the systemic immune‐inflammation index, and napping (model 2). We additionally adjusted for white matter hyperintensity volume and intracranial volume (Brown et al., 2018; Dubost et al., 2019) in persons with valid segmentations (model 3a), or for the AHI (Song et al., 2017) and desaturation rate (model 3b). Second, we investigated PSG‐derived sleep stages (N1, N2, N3, and REM expressed proportional to TST) as determinants, as these may relate differentially to PVS (Berezuk et al., 2015). Third, we restricted analyses to persons without lacunes or cortical infarcts on brain MRI (*n* = 497) to determine the influence of comorbid cerebrovascular disease and reduce potential misclassification of infarcts as PVS (Dubost et al., 2019). Fourth, we restricted analyses to persons with a time interval between sleep and MRI measurement of ≤28 days to assess the influence of the time interval in detecting cross‐sectional associations. Fifth, using the main analysis sample, we investigated non‐linearity in the associations of TST by modelling quadratic terms, as TST can have a U‐shaped relation with poor health outcomes. Lastly, we repeated analyses in the full samples of persons with valid data on either actigraphy and MRI, or PSG and MRI, to reduce selective inclusion due to analysing complete cases only and increase statistical power.

Post hoc, we examined if centrum semiovale‐specific vascular pathology drove associations by additionally adjusting for presence of lobar cerebral microbleeds, indicative of cerebral amyloid angiopathy (Bouvy et al., 2019).

We considered associations at *p* < 0.00198 as statistically significant, to account for multiple testing. This threshold was defined by first Bonferroni‐correcting for testing four brain regions to *p* < 0.0125, and then applying a Sidak correction using the number of effective tests (M_eff_ = 6.45; Galwey, 2009) based on correlations amongst main analysis sleep characteristics.

Missing values on covariates (median 0.2%, ranging from 0% to 9.4%, *n* = 559) were imputed using five multiple imputations with the IBM Statistical Package for the Social Sciences (SPSS®), version 24 (IBM Corp). All analyses were performed with R (package: “glmmADMB”).

## 3 RESULTS

We included 559 participants (mean [*SD*] age 62 [6] years, 52% female; Table 1). The absolute time interval between initiating actigraphy recording and brain MRI scanning was a median (IQR) of 27 (10–65) days; for PSG, this interval was a median (IQR) of 20 (8–45) days. Sleep characteristics were roughly similar between modalities, and median load of the PVS was highest for the centrum semiovale (Table 1). Perivascular space loads across the four brain regions (distributions shown in Figure S1) were not highly correlated (range r*_Spearman_* = 0.12–0.27).

**TABLE 1:** Characteristics of the study population

Characteristic	Study population (*N* = 559)	
	Actigraphy	Polysomnography
Age at sleep measurement, years, mean (*SD*)	62.3 (5.5)	
Female, *n* (%)	288 (52)	
Educational level, *n* (%)		
Primary education	40 (7)	
Lower/intermediate or lower vocational	182 (33)	
Higher or intermediate vocational	159 (29)	
Higher vocational or university	178 (32)	
Smoking status, *n* (%)		
Never	161 (29)	
Former	271 (49)	
Current	127 (23)	
Body mass index, kg/m^2^, mean (*SD*)	27.3 (4.1)	
History of diabetes mellitus, *n* (%)	60 (11)	
History of hypertension, *n* (%)	219 (39)	
History of heart disease, *n* (%)	15 (3)	
Habitual alcohol consumption, g/day, median (IQR)	8 (4–9)	
Systemic immune‐inflammation index^a^, median (IQR)	449 (344–600)	
Daytime napping (number during recording), median (IQR)	1 (0–2)	
White matter hyperintensity volume, cm^3^, median (IQR)	2.3 (1.4–4.3)	
Intracranial volume, cm^3^, mean (*SD*)	1142 (115)	
No lacunes or cortical infarcts on brain MRI, *n* (%)	495 (88)	
Presence of lobar cerebral microbleeds, *n* (%)	55 (9)	
Apnea–Hypopnea Index, events/hr, median (IQR)	12 (5–18)	
Desaturation rate, events/hr, median (IQR)	19 (9–26)	
Sleep durations		
TST, hr, mean (*SD*)	6.2 (0.9)	6.4 (0.9)
N1 (% TST), median (IQR)	NA	12 (9–17)
N2 (% TST), mean (*SD*)	NA	54 (9)
N3 (% TST), median (IQR)	NA	11 (4–19)
REM (% TST), mean (SD)	NA	21 (5)
Sleep onset latency, min, median (IQR)	13 (8–22)	14 (9–22)
Wake after sleep onset, hr, mean (*SD*)	0.9 ± 0.4	1.1 (0.7)
Sleep efficiency, %, median (IQR)	78 (72–83)	83 (78–89)
Absolute time interval sleep–MRI, days, median (IQR)	27 (10–65)	20 (8–45)
Perivascular space load (estimated count), median (IQR)		
Centrum semiovale	6.1 (3.9–9.9)	
Basal ganglia	2.4 (1.8–3.3)	
Hippocampus	2.3 (1.0–4.3)	
Midbrain	1.3 (0.5–2.4)	

IQR, interquartile range; MRI, magnetic resonance imaging; NA, not available; TST, total sleep time.

^a^ Unit = platelet count × granulocyte count/lymphocyte count.

Actigraphy‐estimated higher sleep efficiency was associated with higher PVS load in the centrum semiovale in model 2 (odds ratio [OR] per *SD* increase 1.10, 95% confidence interval [CI] 1.04–1.16, *p* = 0.0008), similarly to model 1 (OR 1.09, 95% CI 1.03–1.15, *p* = 0.003). The association of higher sleep efficiency with higher PVS load in the centrum semiovale remained after additional adjustment for white matter hyperintensity volume and intracranial volume (model 3a: OR 1.10, 95% CI 1.04–1.16, *p* = 0.0008), and for sleep‐disordered breathing characteristics (model 3b: OR 1.10, 95% CI 1.04–1.16, *p* = 0.001). No other actigraphy‐estimated sleep characteristics were associated with the PVS load (Table 2).

**TABLE 2:** Associations of actigraphy‐estimated sleep characteristics with the perivascular space load

Determinants	Rate ratio for association with perivascular spaces load, OR (95% CI)			
	Centrum semiovale	Basal ganglia	Hippocampus	Midbrain
Total sleep time	1.05 (0.99–1.11)	0.97 (0.92–1.03)	0.99 (0.92–1.07)	1.02 (0.94–1.10)
Sleep onset latency	0.91 (0.86–0.97)	1.02 (0.96–1.08)	0.91 (0.84–1.00)	0.97 (0.88–1.06)
Wake after sleep onset	0.96 (0.90–1.02)	1.03 (0.97–1.08)	1.01 (0.93–1.10)	0.98 (0.91–1.06)
Sleep efficiency	**1.10 (1.04–1.16)**	0.99 (0.94–1.05)	1.00 (0.93–1.08)	1.05 (0.97–1.13)

CI, confidence interval; OR, odds ratio.

Estimates are expressed as the relative change in odds of having a higher perivascular spaces load, load per standard deviation increase of the determinant. Estimates were obtained with zero‐inflated negative binomial regression, adjusted for age, sex, education, time interval between measurements of sleep and MRI, smoking status, habitual alcohol consumption, body mass index, presence of hypertension, presence of diabetes mellitus, history of heart disease, systemic immune‐inflammation index, and napping (Model 2). Analyses were performed in the total sample of *n* = 559 participants.

Bold indicates statistical significance after correcting for multiple testing (*p* < 0.00198).

For PSG sleep characteristics, we found no associations with enlarged PVS load in any region (Table 3). Additional analyses also demonstrated no associations of PSG‐derived sleep stages with the PVS loads (Table S1).

**TABLE 3:** Associations of polysomnographic sleep characteristics with perivascular space load

Determinants	Rate ratio for association with perivascular spaces load, OR (95% CI)			
	Centrum semiovale	Basal ganglia	Hippocampus	Midbrain
Total sleep time	1.01 (0.96–1.07)	1.01 (0.95–1.06)	0.99 (0.92–1.07)	1.00 (0.92–1.08)
Sleep onset latency	0.93 (0.86–1.00)	0.96 (0.89–1.04)	0.98 (0.88–1.09)	0.97 (0.87–1.08)
Wake after sleep onset	0.95 (0.89–1.00)	1.00 (0.95–1.06)	1.00 (0.92–1.09)	1.03 (0.95–1.12)
Sleep efficiency	1.07 (1.01–1.13)	1.01 (0.95–1.07)	1.01 (0.93–1.10)	0.99 (0.91–1.07)

CI, confidence interval; OR, odds ratio.

Estimates are expressed as the relative change in odds of having a higher perivascular space load, per standard deviation increase of the determinant. Estimates were obtained with zero‐inflated negative binomial regression, adjusted for age, sex, education, time interval between measurements of sleep and MRI, smoking status, habitual alcohol consumption, body mass index, presence of hypertension, presence of diabetes mellitus, history of heart disease, systemic immune‐inflammation index, and napping (Model 2). Analyses were performed in the total sample of *n* = 559 participants.

Restriction to persons without lacunes or cortical brain infarcts (*n* = 495) did not change our results (Table S2). Post hoc, we further explored the association of sleep efficiency with higher PVS load in the centrum semiovale by additionally adjusting for presence of lobar cerebral microbleeds, which did not change estimates (OR 1.10, 95% CI 1.04–1.16, *p* = 0.0008). After restricting analyses to persons with a time interval between sleep and MRI measurements of ≤28 days (actigraphy *n* = 287; PSG *n* = 349; Table S3), estimates for the association of higher sleep efficiency with higher PVS load in the centrum semiovale were similar, albeit non‐significant after correction for multiple testing (OR 1.12, 95% CI 1.04–1.20, *p* = 0.003).

Repeated analyses in full samples of participants with valid actigraphy and MRI data (*n* = 1228, mean [*SD*] age 65.3 [7.3] years, 51% women), or valid PSG and MRI data (*n* = 769; mean [*SD*] age 63.0 [6.6] years, 54% women), showed similar results as the main analyses (Table S4). We observed a slightly attenuated, but statistically significant, association of higher actigraphy‐estimated sleep efficiency with higher PVS load in the centrum semiovale (OR 1.07, 95% CI 1.03–1.11, *p* = 0.0003).

We further examined potential non‐linearity of associations of actigraphy‐estimated or PSG‐derived TST by modelling quadratic terms. Within the main sample, we found no indications for a non‐linear association (data not shown).

## 4 DISCUSSION

In the present population‐based study, we found that actigraphy‐estimated sleep efficiency was associated with higher PVS load in the centrum semiovale. We found no associations of any other sleep characteristics with higher PVS load in the basal ganglia, hippocampus, or midbrain.

Estimates for sleep efficiency in both measurement modalities were in the same direction, yet stronger and statistically significant when derived from actigraphy. Although less accurate for determining sleep, actigraphy was used over multiple nights and might therefore better indicate habitual sleep compared to a 1‐night PSG assessment. PSG may also be more affected by “first night” effects, despite taking place at home. Possibly, habitual sleep efficiency as captured by actigraphy across multiple days may be more relevant in association with PVS.

Our population‐based findings seem inconsistent with previous clinical studies in patients with sleep or neurological disorders, that predominantly report an association of poor sleep across different characteristics with higher PVS load (Berezuk et al., 2015; Opel et al., 2019; Si et al., 2020; Song et al., 2017; Wang et al., 2020). Noteworthy, two studies from one population‐based cohort also mostly reported null findings (Del Brutto et al., 2019, 2020). A potential difference across study types suggest that an association between sleep and PVS may only exist within the context of clinical disorders. Use of objective measures of sleep versus subjective measures, which have been used in multiple studies (Aribisala et al., 2019; Burnett et al., 2014; Ramirez et al., 2021) and which may capture different aspects of sleep, may also in part explain heterogeneity of study results, including ours.

Regardless, our result indicating an association of higher sleep efficiency with higher PVS load was opposite to what we hypothesised based on previous studies (Aribisala et al., 2019; Berezuk et al., 2015; Del Brutto et al., 2019; Opel et al., 2019; Song et al., 2017; Wang et al., 2020). This could indicate that high sleep efficiency in our present study may not have represented good quality sleep, but rather indicated an insufficient sleep opportunity, accompanied by a high sleep pressure. Although we could not determine whether the habitual sleep opportunity of participants was insufficient, adjusting analyses for napping did not influence the association, nor did we find an association for the proportion of stage N3 or REM sleep, which can be expected to be affected if experiencing an insufficient sleep opportunity. Additionally, we found no relation of TST with PVS load. The association of higher sleep efficiency with higher PVS load is therefore not likely explained by an insufficient sleep opportunity; higher sleep efficiency in our present study likely represents good quality sleep.

Although we do not know why PVS become visible on MRI, factors that may be involved include hypertension, arterial stiffness, inflammation, obstruction of flow, atrophy, or leakage across the blood–brain barrier (Wardlaw et al., 2020). We tried to take these factors into account through covariate adjustment but cannot rule out that they drove the association of sleep efficiency with PVS load. Nevertheless, involvement of abovementioned detrimental factors in the association is not likely considering that most of these are typically associated with poor, not good, sleep (Cuddapah, Zhang, & Sehgal, 2019; Guo et al., 2013; Irwin, Olmstead, & Carroll, 2016; Scullin, 2017).

Equally, it could be hypothesised that PVS, at least in the centrum semiovale, may signify something else than impaired clearance or accumulation of pathology (Brown et al., 2018). Several findings, including ours, seem to indeed support such an alternative interpretation. First, a higher hippocampal load of PVS was associated with better, not worse, memory performance in humans (Hilal et al., 2018). Second, age did not determine PVS load in the centrum semiovale in our cohort, nor did most cardiovascular risk factors (Dubost et al., 2019), all of which are risk factors for brain pathology. Third, adjusting for lobar microbleeds, a marker of cerebral amyloid angiopathy related to more PVS in the centrum semiovale (Bouvy et al., 2019), did not influence our estimates. Fourth, we previously found that higher actigraphy‐estimated sleep efficiency related to less microstructural damage of the white matter in regions overlapping with the centrum semiovale (Kocevska et al., 2019). The relation with such damage, which marks early‐stage cerebral small vessel disease, was opposite to that of the association with PVS in the present study. Together, these and our present findings suggest that PVS in the centrum semiovale in persons from the general population, at least in association with sleep, need not signal pathology per se.

Against the background of aforementioned considerations on interpreting higher sleep efficiency and higher PVS load, we speculate that different mechanisms may explain this association. One explanation is that sleep state‐related increases in fluid flow across PVS (Xie et al., 2013) could enlarge the perivascular compartment, e.g. through repetitively exerting mechanical force. Yet, TST was not related to the PVS load, nor was the proportion of deep sleep in which glymphatic flow has been suggested to be strongest (Hablitz et al., 2019; Xie et al., 2013). Vice versa, our cross‐sectional association may also indicate that the calibre of PVS may affect sleep. A larger calibre of PVS may allow a higher rate of fluid exchange which, assuming that sleep serves among others to clear waste from the brain (Xie et al., 2013), could offer a functional benefit and may increase the efficiency of waste clearance during sleep. In line with this interpretation, a recent study observed an association of functional genetic variation in aquaporin‐4, an astrocytic water channel facilitating flow between PVS and the interstitium, with slow‐wave power during the night in humans (Ulv Larsen et al., 2020). That a large PVS calibre by itself may benefit waste clearance seems also supported by a study using a migraine mouse model, which showed that migraine‐related closure of PVS could impair clearance (Schain, Melo‐Carrillo, Strassman, & Burstein, 2017). Third, sleep efficiency and PVS enlargement underlying detection on MRI may be related through shared biological pathways, e.g. pathways related to astrocytic structure or function (Haydon, 2017; Jessen et al., 2015). Finally, ours may have been a chance finding, suggesting that in the general population sleep is unrelated to PVS load on brain MRI, nor is PVS load a useful marker for sleep‐related waste clearance. Of note, all potential mechanisms may be time invariant as our association was largely independent of whether sleep and PVS were measured with an interval of 28 days or 2 years. Future studies may consider further investigating the association and directionality of sleep efficiency with PVS, and its relevance to glymphatic clearance.

Several methodological considerations deserve mention. First, our algorithm was based on visually rated PVS counts as the “gold standard”. Possibly, morphological aspects or volumes of segmented PVS would better represent subtle differences relevant for glymphatic functioning, although validated automated methods for such segmentation are at present not available. Second, a 1.5‐T MRI field strength has a limited spatial resolution and thus detects only the larger end of the spectrum of PVS. Perivascular spaces detected with higher field strengths, up to 7 T, may better represent physiological aspects of PVS and allow assessment of effects on both very small and large PVS, with a potential relevance to sleep. Third, we could not rule out that the visibility of PVS on MRI may have been influenced by sleeping during MRI acquisition. Falling asleep during MRI scanning is not uncommon and may occur quickly (Tagliazucchi & Laufs, 2014). As sleep has been shown to alter interstitial space volume and fluid exchange in mice (Xie et al., 2013), it may also increase PVS calibre. Although speculative and untested in humans, such effects may have led to overestimation of our association. Fourth, we could not evaluate how sleep related to PVS outside the four pre‐defined regions used in this study. Five, sampling from a population of predominantly European descent may have limited the generalisability of our findings.

We conclude that the association of sleep with PVS in the middle‐aged and elderly population remains limited to that of higher actigraphy‐estimated sleep efficiency with higher PVS load in the centrum semiovale. The direction of this association was opposite to what was expected, and further work is needed to determine the role of sleep in the visibility of PVS on MRI, and the significance of these findings for glymphatic clearance.

## CONFLICT OF INTEREST

All authors (TSL, PY, FD, MAI, MdB, MVW, and AIL) declare no potential competing interests.

## AUTHOR CONTRIBUTION

TSL, MAI, MWV and AIL made substantial contributions to the conception or design of the work. Data was acquired under supervision of MAI, MWV, MdB, and AIL. Data analysis was performed by TSL. All authors (TSL, PY, FD, MAI, MdB, MWV, and AIL) interpreted the data and contributed to drafting the main manuscript text and substantially revising it. Tables were prepared by TSL. All authors approved the final submitted version of this manuscript.

## Supporting information

Supplementary Material

## Data Availability

Data can be obtained on request. Requests should be directed toward the management team of the Rotterdam Study (secretariat.epi@erasmusmc.nl), which has a protocol for approving data requests. Because of restrictions based on privacy regulations and informed consent of the participants, data cannot be made freely available in a public repository.
