# Supplementary material for: Sleep and perivascular spaces in the middle‐aged and elderly population
Source: J Sleep Res. 2021 Sep 22;31(2):e13485. doi: 10.1111/jsr.13485 (PMC9285071; doi:10.1111/jsr.13485)
Supplement: Supplementary file 1 — Supplementary Material [file JSR-31-0-s001.docx]

# Supporting Information

# Supplementary Figure

#
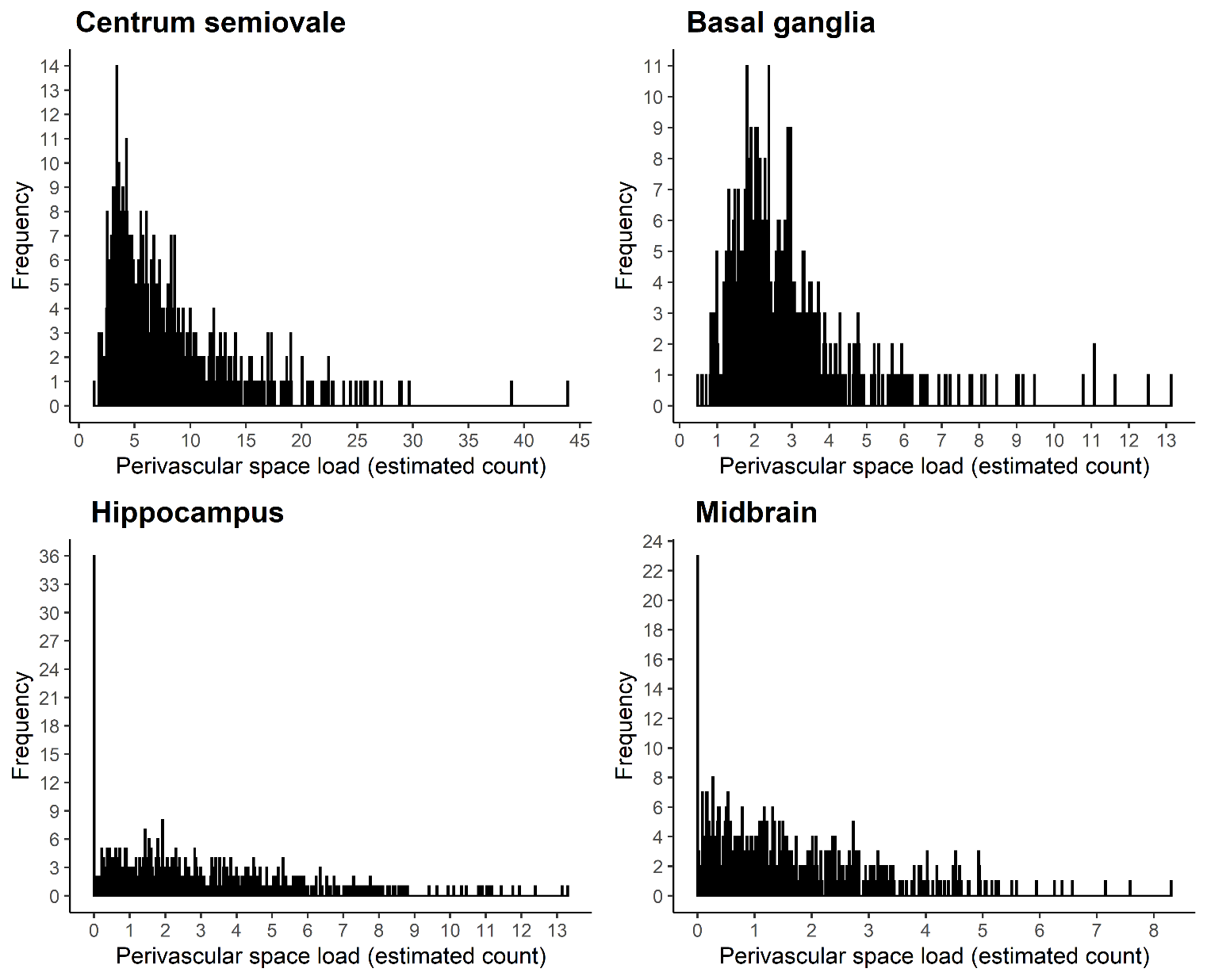


Frequency distributions of perivascular space load, per brain region. Counts could be non-integer due to use of automated methods. Axis intervals differ per plot.

## Supplementary Table 1: Association of separate sleep stages derived from polysomnography with perivascular space load

| Sleep stage duration expressed as | Rate ratio for association with perivascular space load (OR [95% CI]) | | | |
| --- | --- | --- | --- | --- |
| % of total sleep time | Centrum semiovale | Basal ganglia | Hippocampus | Midbrain |
| N1 | 0.97 (0.92-1.03) | 1.02 (0.96-1.08) | 0.97 (0.89-1.06) | 1.06 (0.98-1.15) |
| N2 | 0.99 (0.94-1.04) | 1.00 (0.95-1.05) | 1.01 (0.94-1.09) | 0.97 (0.90-1.04) |
| N3 | 1.02 (0.96-1.08) | 0.99 (0.94-1.05) | 1.01 (0.93-1.09) | 0.99 (0.91-1.07) |
| REM | 1.02 (0.97-1.08) | 1.00 (0.95-1.05) | 0.99 (0.92-1.07) | 1.03 (0.96-1.11) |

Estimates are expressed as the relative change in odds of having a higher perivascular space load, per standard deviation increase of the determinant. We investigated relative sleep stage durations, i.e. as proportion of total sleep time. Estimates were obtained with zero-inflated negative binomial regression, adjusted for age, sex, education, time interval between measurements of sleep and MRI, smoking status, habitual alcohol consumption, body mass index, presence of hypertension, presence of diabetes mellitus, history of heart disease, systemic immune-inflammation index, and napping (Model 2). Analyses were performed in the total sample of n=559 participants.

Abbreviations: CI=Confidence Interval; N=Sample size; OR=Odds ratio.

## Supplementary Table 2: Associations of actigraphy-estimated and polysomnographic sleep characteristics with perivascular space load, restricted to persons without lacunes or cortical brain infarcts on MRI

| Determinants | Rate ratio for association with perivascular space load (OR [95% CI]) | | | |
| --- | --- | --- | --- | --- |
|  | Centrum semiovale | Basal ganglia | Hippocampus | Midbrain |
| Actigraphy |  |  |  |  |
| Total sleep time | 1.07 (1.01-1.14) | 0.98 (0.92-1.04) | 1.02 (0.93-1.11) | 1.01 (0.93-1.10) |
| Sleep onset latency | 0.91 (0.85-0.97) | 1.02 (0.95-1.09) | 0.89 (0.81-0.98) | 0.98 (0.89-1.08) |
| Wake after sleep onset | 0.95 (0.89-1.01) | 1.02 (0.96-1.09) | 0.99 (0.91-1.09) | 0.99 (0.90-1.08) |
| Sleep efficiency | 1.13 (1.06-1.20) | 0.99 (0.93-1.05) | 1.04 (0.96-1.13) | 1.05 (0.96-1.13) |
|  |  |  |  |  |
| Polysomnography |  |  |  |  |
| Total sleep time | 1.02 (0.96-1.08) | 1.01 (0.95-1.08) | 0.98 (0.90-1.07) | 1.00 (0.91-1.09) |
| Sleep onset latency | 0.94 (0.87-1.02) | 0.96 (0.89-1.04) | 0.98 (0.87-1.09) | 0.98 (0.87-1.09) |
| Wake after sleep onset | 0.93 (0.88-1.00) | 0.99 (0.93-1.06) | 1.01 (0.92-1.10) | 1.05 (0.96-1.14) |
| Sleep efficiency | 1.07 (1.01-1.14) | 1.02 (0.96-1.09) | 1.00 (0.91-1.09) | 0.97 (0.89-1.06) |

Estimates are expressed as the relative change in odds of having a higher perivascular space load, per standard deviation increase of the determinant. Estimates were obtained with zero-inflated negative binomial regression, adjusted for age, sex, education, time interval between measurements of sleep and MRI, smoking status, habitual alcohol consumption, body mass index, presence of hypertension, presence of diabetes mellitus, history of heart disease, systemic immune-inflammation index, and napping (Model 2). Analyses were performed in n=495 participants (89% of total).

**Bold** indicates statistical significance after correcting for multiple testing (P<0.00198).

Abbreviations: CI=Confidence Interval; MRI=Magnetic resonance imaging; N=Sample size; OR=Odds ratio

## Supplementary Table 3: Associations of actigraphy-estimated and polysomnographic sleep characteristics with perivascular space load, restricted to persons with a time interval of ≤28 days between sleep and MRI measurements

| Determinants (number of participants | Rate ratio for association with perivascular space load (OR [95% CI]) | | | |
| --- | --- | --- | --- | --- |
| in analyses [% of 559]) | Centrum semiovale | Basal ganglia | Hippocampus | Midbrain |
| Actigraphy (287 [51%]) |  |  |  |  |
| Total sleep time | 1.04 (0.96-1.12) | 0.97 (0.90-1.04) | 1.00 (0.90-1.11) | 1.00 (0.90-1.11) |
| Sleep onset latency | 0.90 (0.83-0.99) | 1.02 (0.94-1.10) | 0.95 (0.85-1.07) | 0.94 (0.83-1.05) |
| Wake after sleep onset | 0.91 (0.84-1.00) | 0.98 (0.91-1.07) | 1.03 (0.91-1.16) | 0.94 (0.83-1.06) |
| Sleep efficiency | 1.12 (1.04-1.20) | 1.01 (0.94-1.08) | 0.98 (0.89-1.09) | 1.01 (0.91-1.12) |
|  |  |  |  |  |
| Polysomnography (349 [62%]) |  |  |  |  |
| Total sleep time | 1.01 (0.94-1.09) | 0.99 (0.93-1.06) | 0.97 (0.88-1.07) | 1.00 (0.91-1.10) |
| Sleep onset latency | 0.88 (0.80-0.98) | 0.93 (0.85-1.03) | 0.95 (0.82-1.10) | 0.91 (0.79-1.05) |
| Wake after sleep onset | 0.95 (0.88-1.03) | 1.02 (0.95-1.10) | 1.04 (0.93-1.17) | 1.03 (0.93-1.14) |
| Sleep efficiency | 1.08 (1.00-1.17) | 1.00 (0.93-1.08) | 0.98 (0.88-1.10) | 1.01 (0.91-1.12) |

Estimates are expressed as the relative change in odds of having a higher perivascular space load, per standard deviation increase of the determinant. Estimates were obtained with zero-inflated negative binomial regression, adjusted for age, sex, education, time interval between measurements of sleep and MRI, smoking status, habitual alcohol consumption, body mass index, presence of hypertension, presence of diabetes mellitus, history of heart disease, systemic immune-inflammation index, and napping (Model 2). Please note that number of participants for analyses restricted on time intervals differed across modalities, as the dates of polysomnography and the start of the actigraphy recording may have differed.

Abbreviations: CI=Confidence Interval; N=Sample size; OR=Odds ratio

## Supplementary Table 4: Associations of sleep characteristics with enlarged perivascular space load, separately for polysomnography and actigraphy in partly overlapping samples

| Determinants (number of participants | Rate ratio for association with perivascular space load (OR [95% CI]) | | | |
| --- | --- | --- | --- | --- |
| in analyses) | Centrum semiovale | Basal ganglia | Hippocampus | Midbrain |
| Actigraphy (N=1,228) |  |  |  |  |
| Total sleep time | 1.04 (1.00-1.08) | 1.04 (1.00-1.08) | 1.02 (0.97-1.08) | 1.01 (0.96-1.06) |
| Sleep onset latency | 0.96 (0.92-1.00) | 1.01 (0.97-1.05) | 0.94 (0.89-1.00) | 1.00 (0.95-1.06) |
| Wake after sleep onset | 0.95 (0.91-0.98) | 1.00 (0.97-1.04) | 0.98 (0.93-1.03) | 0.97 (0.92-1.03) |
| Sleep efficiency | **1.07 (1.03-1.11)** | 1.02 (0.98-1.05) | 1.02 (0.97-1.08) | 1.02 (0.97-1.07) |
|  |  |  |  |  |
| Polysomnography (N=769) |  |  |  |  |
| Total sleep time | 1.02 (0.98-1.07) | 1.02 (0.98-1.07) | 1.00 (0.94-1.06) | 1.01 (0.95-1.08) |
| Sleep onset latency | 0.96 (0.91-1.02) | 0.96 (0.90-1.02) | 1.00 (0.92-1.08) | 1.02 (0.94-1.11) |
| Wake after sleep onset | 0.95 (0.91-1.00) | 1.00 (0.96-1.05) | 1.00 (0.93-1.07) | 0.97 (0.90-1.04) |
| Sleep efficiency | 1.05 (1.00-1.10) | 1.02 (0.97-1.06) | 1.00 (0.94-1.07) | 1.02 (0.95-1.09) |

Estimates are expressed as the relative change in odds of having a higher perivascular space load, per standard deviation increase of the determinant. Estimates were obtained with zero-inflated negative binomial regression, adjusted for age, sex, education, time interval between measurements of sleep and MRI, smoking status, habitual alcohol consumption, body mass index, presence of hypertension, presence of diabetes mellitus, history of heart disease, and systemic immune-inflammation index, and napping (Model 2). For polysomnography-derived determinants, we could not adjust for napping as this was assessed during actigraphy recordings.

**Bold** indicates statistical significance after correcting for multiple testing (P<0.00198).

Abbreviations: CI=Confidence Interval; N=Sample size; OR=Odds ratio
